# Thalidomide combined with transcatheter artierial chemoembolzation for primary hepatocellular carcinoma: a systematic review and meta-analysis

**DOI:** 10.18632/oncotarget.16689

**Published:** 2017-03-29

**Authors:** De-Dong Cao, Hui-Lin Xu, Liang Liu, Yong-Fa Zheng, Si-Fa Gao, Xi-Ming Xu, Wei Ge

**Affiliations:** ^1^ Department of Oncology, RenMin Hospital of WuHan University, WuHan, Hubei, China; ^2^ Department of Oncology, The Fifth Hospital of WuHan, WuHan, Hubei, China; ^3^ Department of Oncology, Fudan University Shanghai Cancer Center, Fudan University, Shanghai, China

**Keywords:** hepatocellular carcinoma, thalidomide, TACE, meta-analysis, survival

## Abstract

**Objective:**

Transcatheter arterial chemoembolization (TACE) and thalidomide have been used for treating primary hepatocellular carcinoma(HCC). This study aims to evaluate the clinical efficacy and safety of thalidomide and TACE in primary HCC.

**Methods:**

Randomized controlled trials(RCTs) about efficacy and safety of thalidomide combined with TACE for primary HCC were identified from the Cochrane Library, Pubmed, Embase, CNKI, and Wan Fang until August, 2016. The retrieved trials were reviewed and the data were extracted by two reviewers, independently. Combined analyses of survival rates, overall response rate(ORR), disease control rate(DCR), changes of KPS, parameters of cellular immunity and vascular endothelial growth factor(VEGF), and adverse events were performed using RevMan 5.3 software.

**Results:**

A total of 23 RCTs involving 1836 patients were included. The results showed that thalidomide plus TACE was significantly superior in increasing 6-month survival rate(OR=1.79, 95% CI:1.02-3.15, *P*=0.04), 1-year survival rate(OR=1.76, 95% CI:1.38-2.24, *P*<0.0001), 1.5-year survival rate(OR=4.72, 95% CI:2.64-8.43, *P*<0.001), 2-year survival rate(OR=1.78, 95% CI:1.37-2.30, *P*<0.001), ORR(OR=1.89, 95% CI:1.48-2.42, *P*<0.0001), DCR(OR=2.62, 95% CI:1.90-3.63, *P*<0.001), improvement in cellular immunity(MD=0.63, 95% CI:0.45-0.80, *P*<0.0001), and reduction of VEGF(MD=-119.71, 95% CI:-135.75—103.68, *P*<0.0001), when compared with TACE group. The incidences of gastrointestinal reactions, myelosuppression, and liver dysfunction were similar between combination group and TACE group(*P*>0.05). However, compared to TACE, the combination of thalidomide and TACE had a higher incidence of drug rash(OR=6.35, 95% CI:2.75-14.68, *P*<0.0001).

**Conclusion:**

Our findings suggest that thalidomide combined with TACE shows better clinical efficacy and tolerable adverse events in patients with primary HCC when compared with TACE alone.

## INTRODUCTION

Hepatocelluar carcinoma(HCC) is a primary malignant disease derived from liver cells. HCC is one of the most common digestive cancers worldwide and the third most common cause of cancer related death in the Asia-Pacific region [1]. In China, HCC is the second most common cancer after lung cancer. The treatments options for HCC includes hepatectomy, liver transplantation, local ablative therapy, chemotherapy and molecular targeted therapies [2]. The hepatectomy and liver transplantation are considered to be the curative therapies, however, HCC is usually diagnosed at advanced stage when the application of curative treatments seems to be of little value [3, 4]. For intermediate HCC identified by the Barcelona Clinic Liver Cancer (BCLC) staging system, the local-regional therapies including trans-catheter arterial chemoembolization (TACE), radiofrequency ablation (RFA), and percutaneous ethanol injection (PEI) are suggested as the optimal treatments [5, 6]. These methods have been shown to prolong survival and treatment response for patients, particularly TACE [7–9].

TACE delivers chemotherapeutic agents to the cancer location while blocking the blood supply supporting the growth of tumor [10]. However, the 3-year survival rate is only about 20% due to the complex recurrence mechanisms of HCC. One of them is believed to be angiogenesis caused by vascular endothelial growth factor(VEGF) [11]. It is reported that the elevated levels of VEGF and other angiogenic factors secreted by the hypoxic tumor cells after TACE has the role of promoting abnormal angiogenesis [11]. Indeed, by targeting VEGF, platelet derived growth factor receptor, and other signaling, anti-angiogenesis agents proved to block tumor development and neo-angiogenesis [12, 13].

In recent years, several molecular targeted agents inhibiting the process of angiogenesis have been introduced into clinical practice and show effective results [14]. Thalidomide is not only capable of inhibiting angiogenesis, but also modulating immunity [15, 16]. Thalidomide alone or in combination with other regimen has been widely used in treating cancers such as multiple myeloma [17, 18], HCC [4, 19], lung cancer [20], and bladder cancer [21]. With regards to HCC, many clinical retrospective studies or RCTs [3, 19, 22–29] evaluated the effect of adding thalidomide to TACE on patients with primary HCC, and the results of most of these studies are positive. Patients treated with thalidomide and TACE have a better clinical response and survival than TACE alone. However, these evidences are lack of powerful convincing as the samples of these studies are relatively small. Besides, whether the use of thalidomide in combination with TACE has a better clinical efficacy in patients with intermediate or advanced HCC, there is no final conclusion.

With the concern mentioned above, we systematically identified clinical studies on the topic of thalidomide with TACE for treating HCC, extracted data and conducted this meta-analysis to evaluate the efficacy and safety of thalidomide when combined with TACE, aimed to provide evidence for supporting administration of thalidomide in HCC patients treated with TACE.

## RESULTS

### Results of literature research and baseline characteristics of included studies

**Figure 1:**
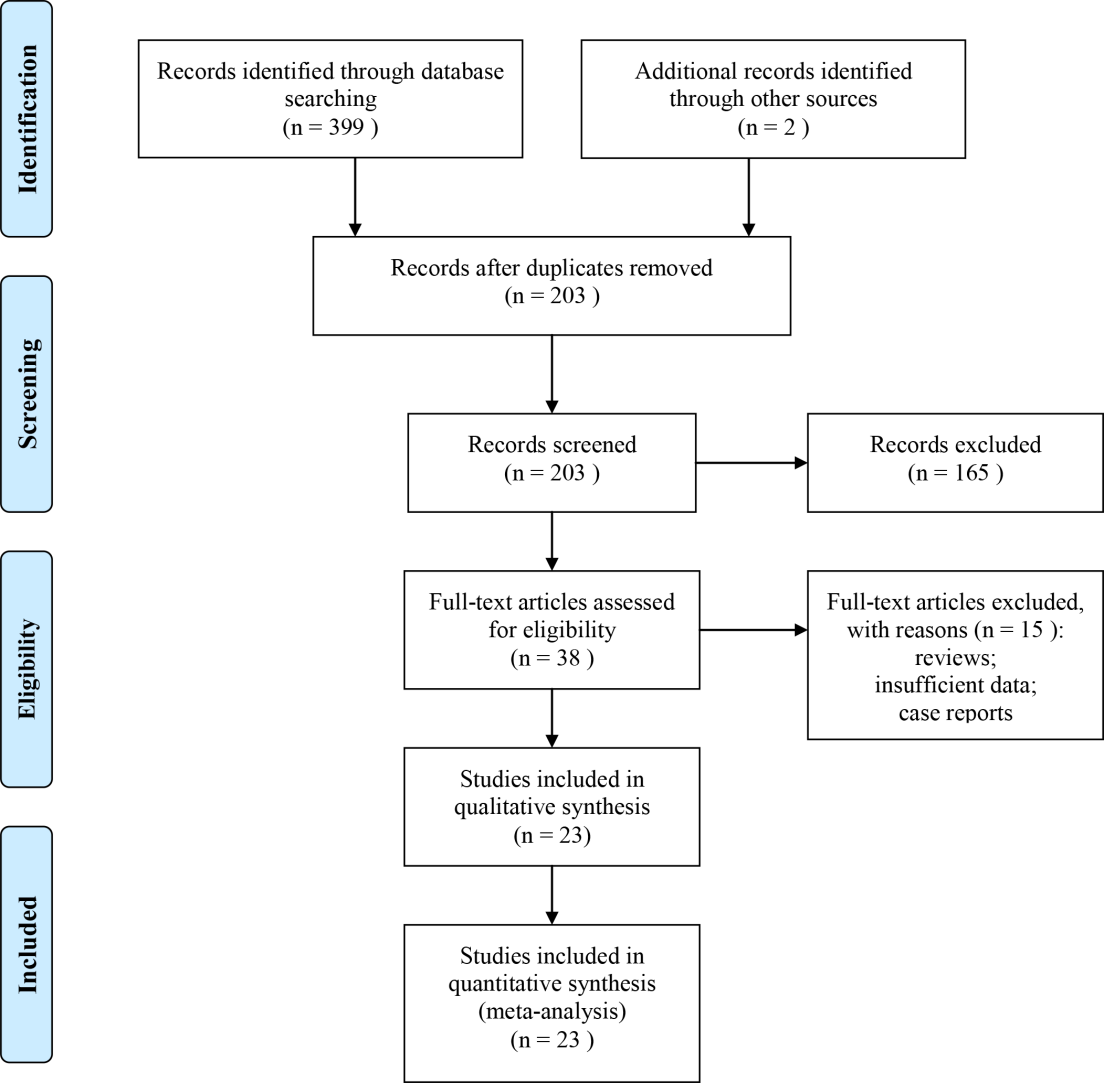
The flow chart of systematically research for eligible studies

After systematically retrieving in selected databases, a total of 401 articles were identified. 203 records were left after duplicates removed. 165 of them were discarded after reviewing the title and abstract. The full-text of the left 38 studies were further reviewed and 15 of them were excluded. Finally, 23 clinical studies [30–52] including 1836 patients with primary HCC were included. There were 904 cases in the thalidomide combined with TACE group and 932 cases in the TACE group. The overall process of study selection is presented in Figure 1.

The baseline characteristics of included studies are listed in Table 1. Most of the eligible studies did not report the method of blinding. All of the studies were on the topic of efficacy and safety of thalidomide combined with TACE versus TACE alone in intermediate or advanced primary HCC patients. The age and sex distribution were similar in both groups. All of the included trials were performed in China. The dose of thalidomide and cycle of TACE were in accordance within these studies. The regimen of TACE were similar between different studies. The studies of Yun-xiao Lin, et al. [36, 40] and Chang-nan Chen, et al. [36] reported more cases than other studies. All of the included studies reported the therapeutic effects of thalidomide according to the mRECIST criteria. The ORR and DCR were defined as (cases of CR plus PR)/total cases and (cases of CR, PR, and SD)/total cases, respectively. All of the studies reported ethics and written consent during treatments. The concentrations of VEGF and AFP were reported to be tested by Enzyme-linked immuno sorbent assay(ELISA) test in included studies. The parameters of cellular immunity were tested by flow cytometry and analyzed by CellQuest in included studies.

**Table 1:** Baseline characteristics of included studies

Studies	N		Sex		Age		Interventions		Outcomes
	T	C	Male	Female	T	C	T	C	
Wei-sheng Zheng-1, 2007	15	15	27	3	21-70	21-69	TACE+TLD: TLD, 200mg, po, qd	TACE	QOL, IF, TNF-a,
Wei-sheng Zheng-2, 2007	15	15	27	3	21-70	21-69	TACE+TLD: TLD, 200mg, po, qd	TACE	QOL, IF, TNF-a,
Hai-lan Lin, 2007	32	31	53	10	22-75	22-75	TACE+TLD: TLD, 200mg, po, qd	TACE	VEGF
Ming-zhi Hao, 2007	45	51	86	10	22-75	22-75	TACE+TLD: TLD, 200mg, po, qd	TACE	SR
Xiao-jun Qi, 2007	34	34	48	20	30-83	30-83	TACE+TLD: TLD, 200mg, po, qd	TACE	VEGF
Xiu-fang Liu, 2007	40	58	62	36	36-70	36-70	TACE+TLD: TLD, 200mg, po, qd	TACE	ORR, DCR, SR, AE
Chang-nan Chen, 2008	62	60	106	16	18-82	18-82	TACE+TLD: TLD, 200mg, po, qd	TACE	ORR, DCR, SR, AE
Long Feng, 2008	15	20	28	7	31-73	31-73	TACE+TLD: TLD, 200mg, po, qd	TACE	ORR, DCR, SR,
Gao-hua Han, 2008	18	18	29	7	29-76	34-68	TACE+TLD: TLD, 200mg, po, qd	TACE	ORR, DCR, AFP, VEGF, IF
Wei-min Wang, 2009	21	26	30	17	25-75	25-75	TACE+TLD: TLD, 200mg, po, qd	TACE	ORR, DCR, AE
Xiao-bing Yuan, 2009	18	21	31	8	35-75	35-75	TACE+TLD: TLD, 200mg, po, qd	TACE	SR
Hai-ying Jiang, 2010	50	50	77	23	30-60	30-60	TACE+TLD: TLD, 200mg, po, qd	TACE	ORR, DCR, SR, VEGF, AE
Yun-xiao Lin, 2010	70	60	94	26	31-77	30-76	TACE+TLD: TLD, 200mg, po, qd	TACE	SR
Fei Wang, 2010	38	34	48	24	15-70	15-70	TACE+TLD: TLD, 200mg, po, qd	TACE	SR, VEGF
Zeng-hu Zhao, 2010	45	42	66	21	39-68	39-68	TACE+TLD: TLD, 200mg, po, qd	TACE	ORR, DCR, VEGF, AFP
Yan Shang, 2011	60	60	69	51	31-74	31-74	TACE+TLD: TLD, 200mg, po, qd	TACE	ORR, DCR, SR, AE
Hai-ying Jiang, 2011	50	50	77	23	37-72	37-72	TACE+TLD: TLD, 200mg, po, qd	TACE	ORR, DCR, SR, AE
Zhen-kai Ye, 2013	38	39	72	5	23-69	23-69	TACE+TLD: TLD, 200mg, po, qd	TACE	ORR, SR
Ji-qun Pan, 2013	27	27	38	16	36-76	36-76	TACE+TLD: TLD, 200mg, po, qd	TACE	ORR, DCR, VEGF, AFP
Xiang-dong Lu, 2014	30	30	41	19	26-77	26-77	TACE+TLD: TLD, 200mg, po, qd	TACE	ORR, VEGF
Xia Zhang, 2015	50	50	68	32	35-73	35-73	TACE+TLD: TLD, 100-200mg, po, qd	TACE	IF
Di-yang Xie, 2015	42	48	NA	NA	NA	NA	TACE+TLD: TLD, 150-400mg, po, qd	TACE	SR, AE
Cheng Zhang, 2015	40	40	47	33	31-77	30-76	TACE+TLD: TLD, 200-300mg, po, qd	TACE	ORR, DCR, SR, AE
Kang Zheng, 2016	49	53	74	28	49-62	47-62	TACE+TLD: TLD, 200mg, po, qd	TACE+sorafenib: Sorafenib, 400mg, po, bid	ORR, DCR, SR, AFP, AE

Abbreviation: NA: not available; po, oral administration; tid, three times a week; QOL, quality of life; PRS, Patient-Reported Symptoms; IF, immunity function; T, treatment; C, control; TLD, thalidomide; TACE, transcatheter arterial chemoembolization; SR, survival rate; ORR, overall response; DCR, disease control rate; AE, adverse events;

### Results of quality evaluation

The overall quality of included studies was evaluated according to criteria for bias risk assessment in the Cochrane collaboration handbook 5.1.4. All of the eligible trials reported the application of randomization. Among these studies, four [32, 33, 44, 46] of them mentioned about the method of randomized number table, one [47] mentioned the use of envelop method, and the rest of them did not report detailed method of randomization. All of the studies did not report whether the treatment regimen was allocated or not. Only two studies [38, 39] used blinding method in the process of treatments. All of the studies presented the data about baseline characteristics and endpoints of participants and they were regarded as reporting complete data. Other sources of bias were not evaluable as all of the studies did not provide enough bias related data. The detailed information about quality assessment in each study is presented in Table 2.

**Table 2:** methodological quality evaluation of included studies

Studies	Randomization	Allocation	Blinding	Incomplete data	Selective reporting	Other bias
Wei-sheng Zheng-1, 2007	Y	N	N	N	N	NR
Wei-sheng Zheng-2, 2007	Y	N	N	N	N	NR
Hai-lan Lin, 2007	Y	N	N	N	N	NR
Ming-zhi Hao, 2007	Y	N	N	N	N	NR
Xiao-jun Qi, 2007	Y	N	N	Y	N	NR
Xiu-fang Liu, 2007	Y	N	N	N	N	NR
Chang-nan Chen, 2008	Y	N	N	Y	N	NR
Long Feng, 2008	Y	N	N	N	N	NR
Wei-min Wang, 2009	Y	N	Y	N	N	NR
Xiao-bing Yuan, 2009	Y	N	Y	N	N	NR
Fei Wang, 2010	Y	N	N	N	N	NR
Zeng-hu Zhao, 2010	Y	N	N	N	N	NR
Hai-ying Jiang, 2010	Y	N	N	N	N	NR
Yun-xiao Lin, 2010	Y	N	N	Y	N	NR
Yan Shang, 2011	Y	N	N	N	N	NR
Hai-ying Jiang, 2011	Y	N	N	N	N	NR
Zhen-kai Ye, 2013	Y	N	N	N	N	NR
Ji-qun Pan, 2013	Y	N	N	N	N	NR
Xiang-dong Lu, 2014	Y	N	N	N	N	NR
Xia Zhang, 2015	Y	N	N	N	N	NR
Di-yang Xie, 2015	Y	N	N	Y	N	NR
Cheng Zhang, 2015	Y	N	N	N	N	NR
Kang Zheng, 2016	Y	N	N	N	N	NR

Abbreviation: Y, yes; N, no; NA, not available; NR, not reported

### Results of Meta-analysis

#### Survival rates

**Figure 2:**
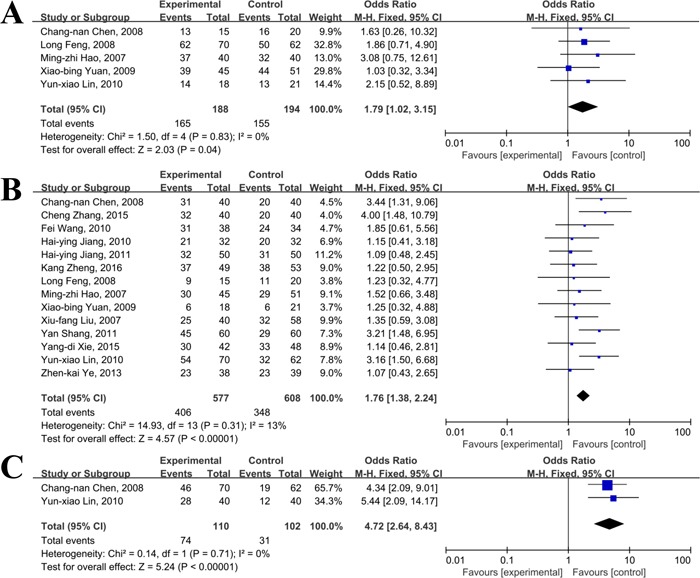
Meta-analyses of 6 (**A**), 12 (**B**), and 18 (**C**) months survival rates between thalidomide combined with TACE versus TACE alone in patients with primary HCC.

**Figure 3:**
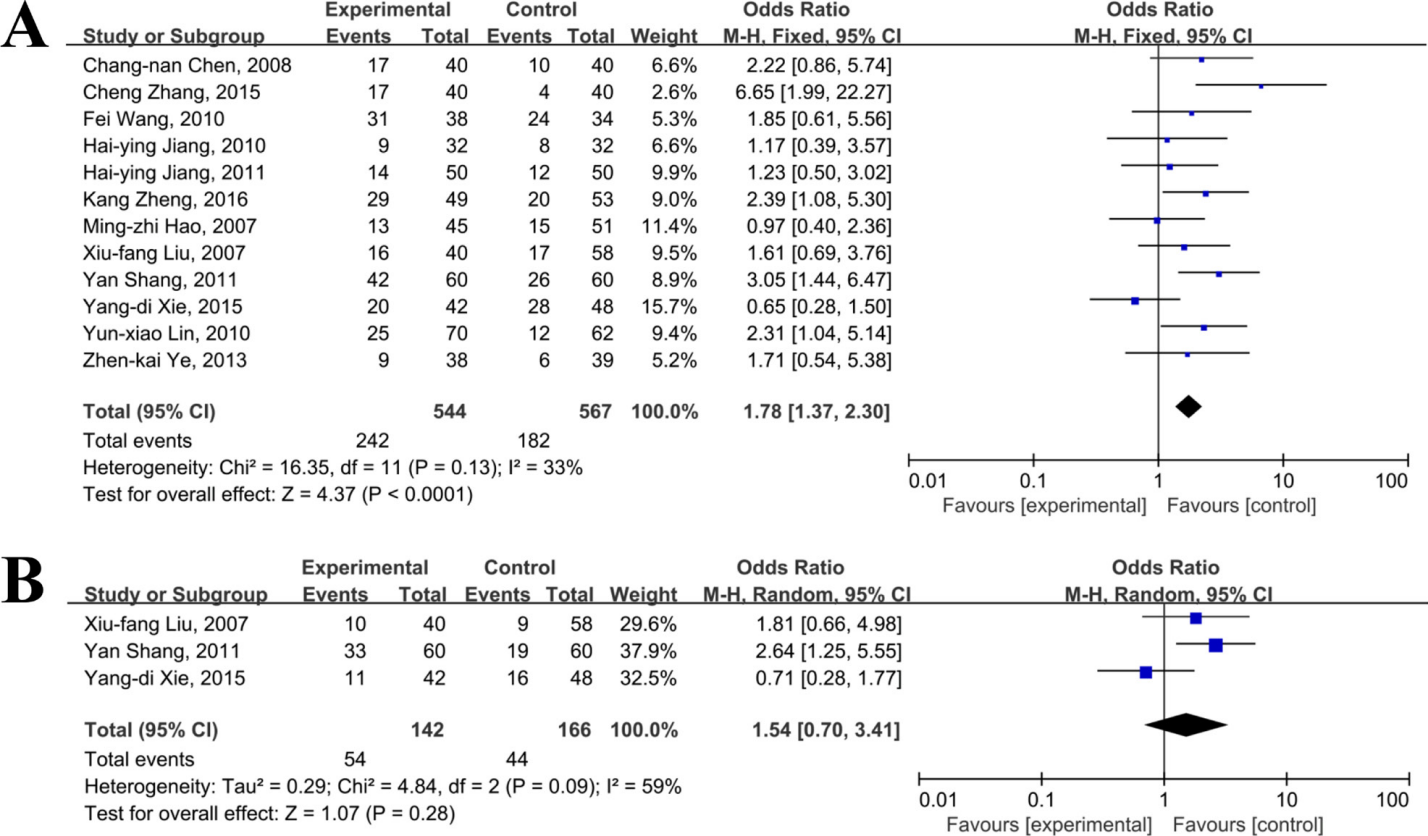
Meta-analyses of 24 (**A**) and 36 (**B**) months survival rates between thalidomide combined with TACE *versus* TACE alone in patients with primary HCC.

There were 5 [32, 35, 36, 40, 53], 14 [32, 34–38, 40, 42–45, 47, 50, 51], 2 [36, 40], 12 [32, 34, 36, 37, 40, 42–45, 47, 50, 51], and 3 [34, 45, 51] studies reported data of survival rates at 6, 12, 18, 24, 36 months, respectively. As shown in Figure 2 and 3, there were no significant heterogeneity in most of the combined analyses in terms of survival rates, and the fixed effect model was used, except the 36 months survival. The results of meta-analyses revealed that compared to TACE alone, primary HCC patients treated with thalidomide and TACE could gain significantly superior in increasing 6-month survival rate(OR=1.79, 95% CI:1.02-3.15, *P*=0.04), 1-year survival rate(OR=1.76, 95% CI:1.38-2.24, *P*<0.0001), 1.5-year survival rate(OR=4.72, 95% CI:2.64-8.43, *P*<0.001), and 2-year survival rate(OR=1.78, 95% CI:1.37-2.30, *P*<0.001), respectively. However, there was no significant difference between combination group and TACE alone group in 3-year survival rate(OR=1.54, 95% CI:0.70-3.41, *P*=0.09).

### Overall response rate

**Figure 4:**
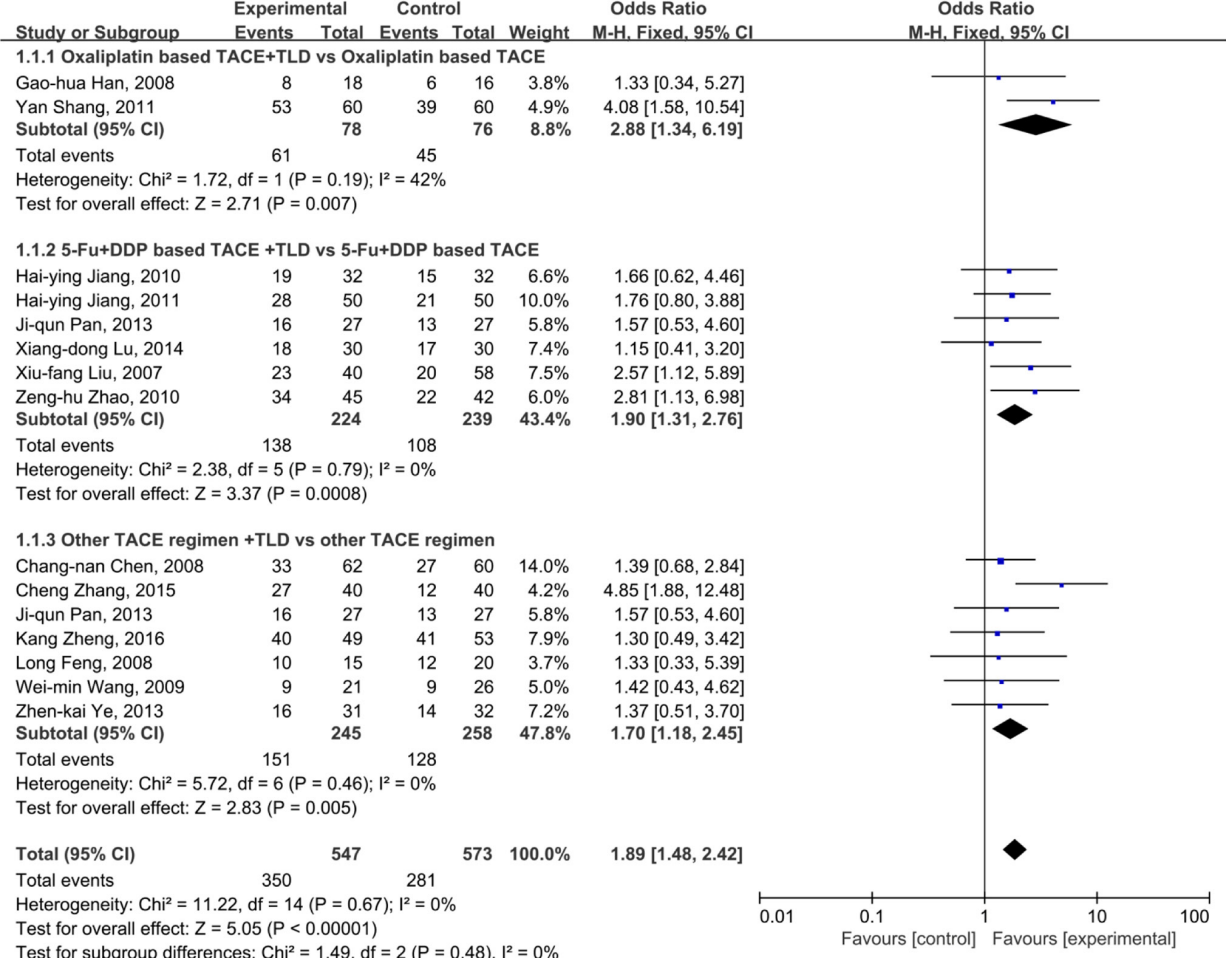
Comparison of ORR between thalidomide combined with TACE *versus* TACE alone in patients with primary HCC

Of the 23 included studies, 15 [34–37, 39, 41, 42, 44–47, 49–51] of them reported the ORR in primary HCC patients receiving thalidomide and TACE versus TACE alone. After inputting effective numbers and total cases in RevMan 5.3 software, the combined results showed that there was a low risk of heterogeneity as indicated by the I^2^ value=0%, and the fixed model was used to calculate the Odds Ratio(OR). As illustrated by Figure 4, there were 350(63.9%) cases gained significantly improvements in ORR in patients treated with thalidomide and TACE, when compared to 281 cases(49.0%) in TACE group(OR=1.89, 95%CI: 1.48-2.42; *P*<0.01). This finding indicated that thalidomide combined with TACE had a better efficacy than TACE alone in primary HCC patients. The sensitivity analyses showed that the final effect of combination regimen was reliable.

We introduced sub-group analysis to evaluate the clinical efficacy of thalidomide combined with different regimen of TACE. Included studies were divided into 3 groups(oxaliplatin based regimen, 5-Fu+DDP based regimen, and 5-Fu+ADM\EPI based regimen), according to the agents used in the TACE process. By using fixed effect model, the combined results showed that thalidomide plus oxaliplatin based regimen had a higher OR(OR=2.88, 95%CI: 1.34-6.19; *P=*0.007 ), followed by 5-Fu+DDP based regimen(OR=1.90, 95%CI: 1.31-2.76; *P<*0.001) and 5-Fu+ADM\EPI based regimen (OR=1.87, 95%CI: 1.21-2.88; *P=*0.005).

### Disease control rate

**Figure 5:**
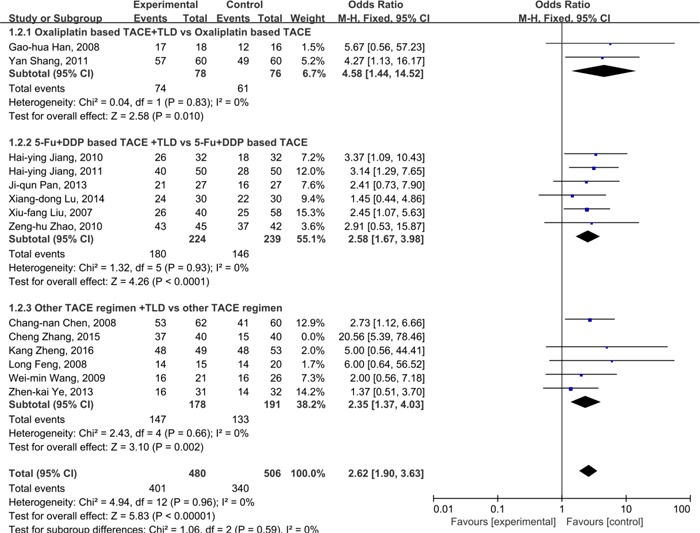
Comparison of DCR between thalidomide combined with TACE *versus* TACE alone in patients with primary HCC

There were 13 studies [34–37, 39, 41, 42, 44–47, 49, 50] reported data of disease control rate(DCR) with regards to regimen of thalidomide combined with TACE versus TACE alone in patients with primary HCC. As indicated by the *I*^2^%=0%, it was considered that there was no significant heterogeneity across included studies, and the fixed model was applied. As illustrated in Figure 5, the combined data showed that the DCR in thalidomide plus TACE group was significantly better than that in the TACE group(83.5% vs. 67.2%; OR=2.62, 95%CI: 1.90-3.63; *P*<0.01), suggesting that patients treated with thalidomide plus TACE may have a better DCR than TACE alone.

### Quality of life

Only 1 of the included studies reported data about quality of life in terms of KPS scores. However, the meta-analysis was not performed as there was insufficient data and significant heterogeneity in the selected study. As shown by the study of Wei-sheng Zheng, et al., [30] compared to TACE treatment, a significant improvement in KPS scores was achieved in patients receiving thalidomide plus TACE treatment(KPS score: 51.71±8.75 vs. 43.13% ±7.39; *P*<0.05), showing that thalidomide plus TACE may have a better effect in improving the quality of life in patients with primary HCC when compared with TACE treatment.

### Parameters of cellular immunity

**Figure 6:**
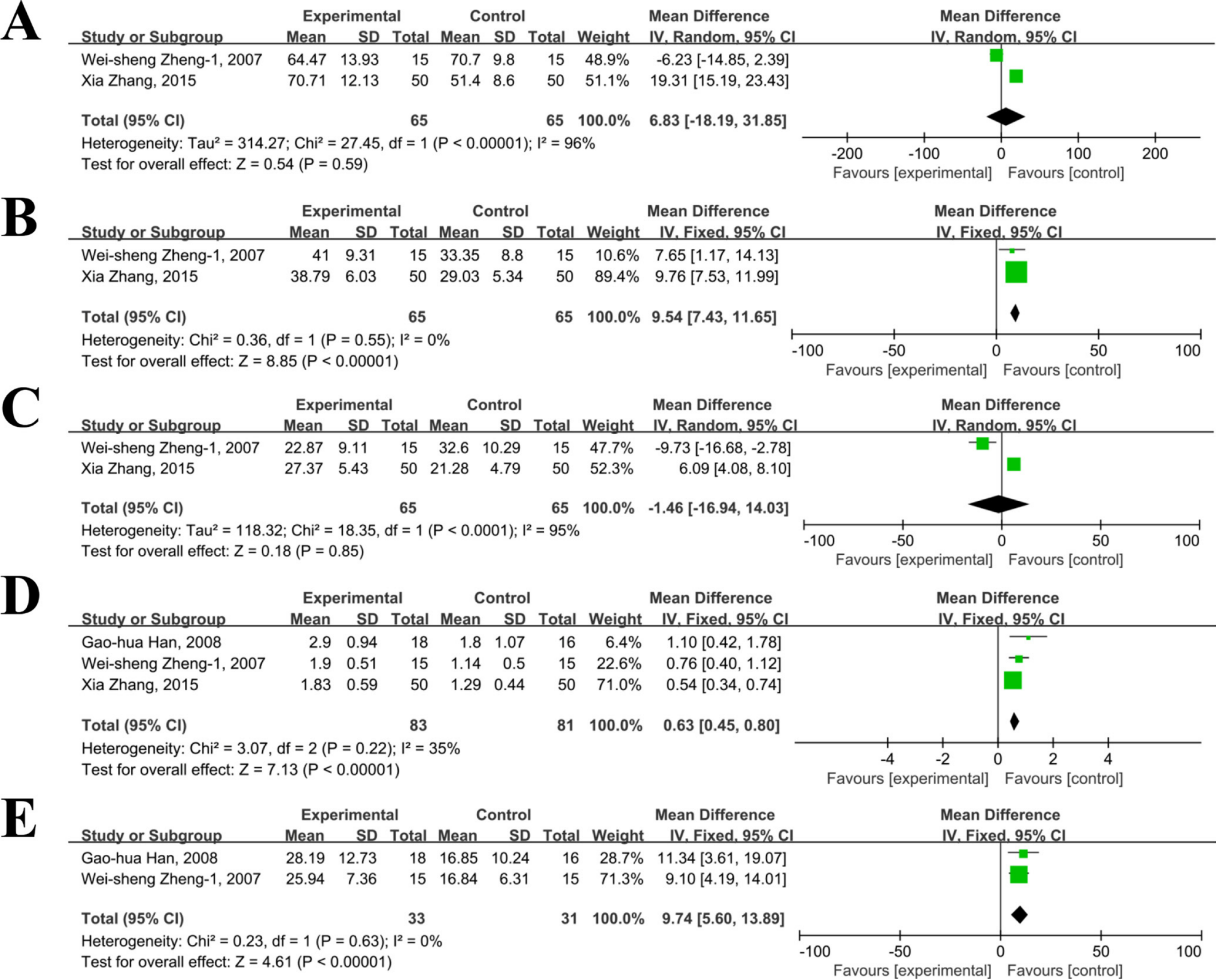
Comparison of cellular immunity between thalidomide combined with TACE *versus* TACE alone in patients with primary HCC (**A**, CD_3_^+^; **B**, CD_4_^+^; **C**, CD_8_^+^; **D**, CD_4_^+/^CD_8_^+^; **E**, NK.).

Only 3 [37, 48, 52] of the included studies reported data about parameters of cellular immunity including CD_3_^+^, CD_4_^+^, CD_8_^+^, CD_4_^+^/CD_8_^+^, and NK. The results of meta-analysis revealed that significant heterogeneity was observed within these two trials(*I*^2^%>50%) on CD_3_^+^ and CD_8_^+^, and the random effect model was used to synthesize the data. As shown in Figure 6, compared to TACE treatment, significant improvements in CD_4_^+^(MD=9.54, 95%CI: 7.43-11.65; *P*<0.01), CD_4_^+^/CD_8_^+^(MD=0.63, 95%CI: 0.45-0.80; *P*<0.01), and NK(MD=9.74, 95%CI: 5.60-13.89; *P*<0.001) were achieved in patients receiving thalidomide plus TACE treatment, showing that thalidomide plus TACE may have a better effect in improving the quality of life in patients with primary HCC when compared with TACE treatment. However, the percentages of CD_3_^+^(MD=6.83, 95%CI: -18.19-31.85; *P=*0.59), and CD_8_^+^(MD=-1.46, 95%CI: -16.94-14.03; *P*=0.85) were not significantly different in combination group and TACE group.

### Changes of VEGF and AFP

**Figure 7:**
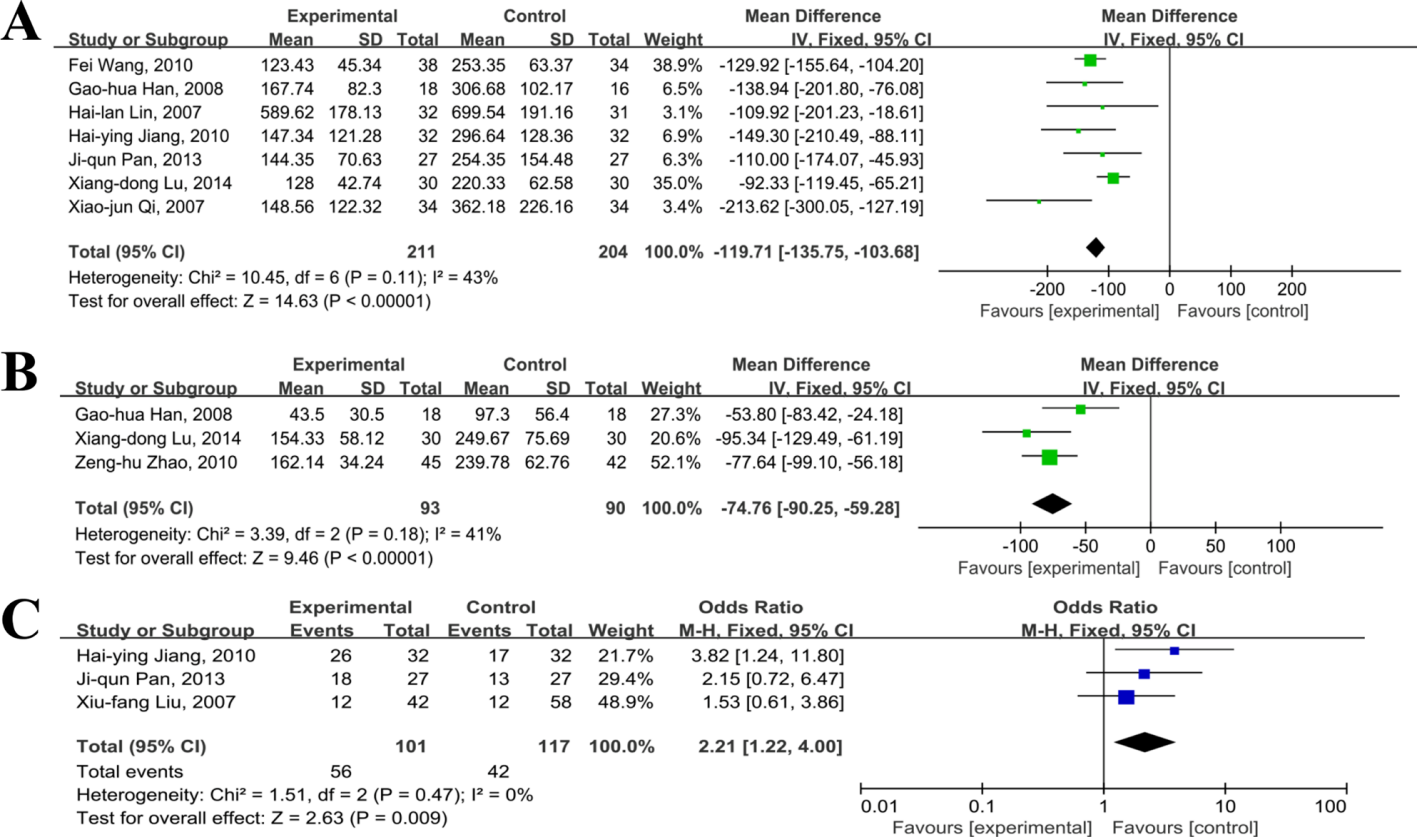
Comparison of changes of VEGF and AFP between thalidomide combined with TACE *versus* TACE alone in patients with primary HCC (**A**, changes of VEGF; **B**, changes of AFP; **C**, AFP reduction rates).

With regards to VEGF, 7 [33, 37, 42, 43, 46, 49, 54] of 23 RCTs provided data of changes of VEGF in patients treated with thalidomide and TACE. There was moderate heterogeneity(I^2^=43%) between these selected studies, so the fixed effect model was introduced. As shown in Figure 7, the levels of VEGF were significantly lower in patients treated with thalidomide and TACE when compared to TACE alone(MD=-119.71, 95% CI:-135.75--103.68; *p*<0.01), suggesting that thalidomide may have role of decreasing the level of VEGF when combined with TACE.

Six studies [34, 37, 41, 42, 46, 49] provided data about changes of AFP level or numbers of AFP reduction in patients receiving thalidomide combined with TACE versus TACE. For AFP levels, the analysis of heterogeneity showed that there was low risk of heterogeneity across selected studies(*I*^2^%=41%), and the fixed effect model was used. As presented in Figure 7, the combined mean difference was -74.76(95%CI:-90.25, -59.28; *p*<0.001). This suggested that thalidomide in combination with TACE had a better effect on reducing concentration of AFP in serum than TACE alone. For numbers of AFP reduction, three studies reported data of decrease rates of AFP level in patients with primary HCC receiving TACE plus thalidomide. As the *I*^2^%=0%, it was considered that there was no significant heterogeneity across included studies, and the fixed effect model was applied during the process of analysis. As shown in Figure 7, the cases with decreased AFP was 56 in combination group, and it was 42 cases in TACE alone group(55.4% vs. 35.9%; OR=2.21, 95%CI: 1.22-4.00; *P*=0.009), suggesting that the combination regimen had a better effect on decreasing AFP.

**Table 3:** Detailed information of TACE regimen in each included studies

Studies	TACE regimens
Wei-sheng Zheng-1, 2007	5-Fu 1.0g, OXA 200mg
Wei-sheng Zheng-2, 2007	5-Fu 1.0g, OXA 200mg
Hai-lan Lin, 2007	5-Fu 0.5-1.0g, OXA 100-200mg, Gem 0.4-1.6g
Ming-zhi Hao, 2007	5-Fu 0.5-1.0g, OXA 100-200mg, Gem 0.4-1.6g
Xiao-jun Qi, 2007	5-Fu 1.0g, HCPT 20mg, DDP 100mg
Xiu-fang Liu, 2007	5-Fu 0.75-1.0mg, DDP 80-100mg, ADM 40-50mg
Chang-nan Chen, 2008	NR
Long Feng, 2008	5-Fu 1.0g, MMC 8-12mg, ADM 40-60mg \ EPI 70-90mg
Gao-hua Han, 2008	5-Fu 750mg/m^2^, ADM 30-40mg/m^2^, OXA 125mg/m^2^
Wei-min Wang, 2009	5-Fu 600mg/m^2^, HCPT 20mg/m^2^, EPI 60 mg/m^2^
Xiao-bing Yuan, 2009	5-Fu 600mg/m^2^, HCPT 20mg/m^2^, EPI 60 mg^/^m2
Hai-ying Jiang, 2010	5-Fu 0.8-1.2g, DDP 80-100mg, EPI 80-120mg
Yun-xiao Lin, 2010	NR
Fei Wang, 2010	OXA 100mg, DOX 20mg
Zeng-hu Zhao, 2010	5-Fu 1.0g, DDP 60-80mg, HCPT 10-20mg
Yan Shang, 2011	OXA 150mg, EPI 70-90mg, HCPT, 10mg
Hai-ying Jiang, 2011	5-Fu 0.8-1.2g, DDP 80-100mg, EPI 80-120mg
Zhen-kai Ye, 2013	Gem 0.8-1.4g
Ji-qun Pan, 2013	5-Fu 0.75-1.0g, DDP 80-100mg, EPI 80-120mg
Xiang-dong Lu, 2014	5-Fu 0.75-1.0g, DDP 80-100mg, EPI 80-120mg
Xia Zhang, 2015	5-Fu 0.75-1.25g, DDP 80-120mg, OXA 200mg, EPI 80-140mg
Di-yang Xie, 2015	OXA, 100-150mg, 5-Fu 1.0, MMC 10mg
Cheng Zhang, 2015	5-Fu 1.0g, ADM 40mg/m^2^, MMC 10mg/m2
Kang Zheng, 2016	ADM 20-50mg

Abbreviation: 5-Fu, 5-fluoro-2,4(1h, 3h)pyrimidinedione; ADM, Adriamycin; MMC, Mitomycin; OXA, Oxaliplatin; DDP, Cisplatin; EPI, Epirubicin; HCPT, Hydroxycamptothecine; Gem, Gemcitabine; NR, not reported; DOX, Doxorubicin.

### Adverse events

**Figure 8:**
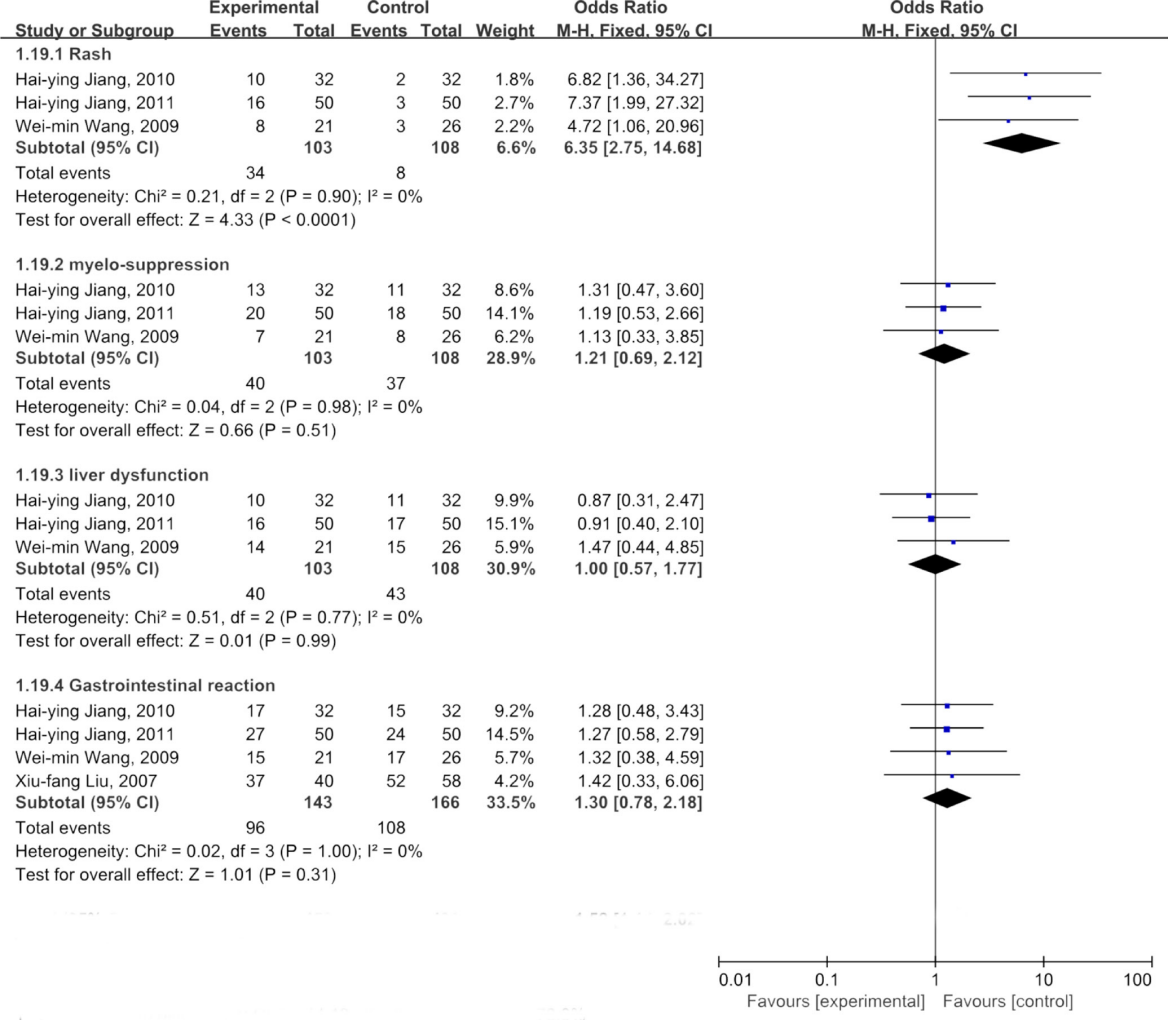
Comparison of common adverse events between thalidomide combined with TACE *versus* TACE alone in patients with primary HCC

The common adverse events in the included studies [34, 39, 42, 44] were classed into hematological and non-hematological events(Figure 8). With regards to hematological toxicities, three studies [39, 42, 44] reported incidences of myelosuppression. The meta-analysis was performed based on the fixed effect model as there was no significant heterogeneity(*I*^2^%<10%). The results showed that the incidences of myelo-suppression were similar between thalidomide plus TACE group and TACE alone, and there was no significantly difference (OR=1.21, 95%CI:0.69-2.12; *P=*0.51). Non-hematological toxicities were mainly exhibited as drug rash, liver dysfunction, and gastrointestinal reactions. Three studies [39, 42, 44] provided events of drug rash and the detection of heterogeneity demonstrated negative finding (*I*^2^%=0%). The results of meta-analysis based on the fixed effect model revealed that there was significant difference in the incidence of drug rash in thalidomide plus TACE group when compared with TACE, indicating that the incidence of drug rash was higher in the combination group than that in the TACE alone group (OR=6.35, 95%CI: 2.75-14.68; *P*<0.01). Four studies [34, 39, 42, 44] reported the occurrence of gastrointestinal reactions. We used fixed effect model to calculate the combined result of gastrointestinal reactions, and the results showed that there was no statistically significant difference between thalidomide in combination with TACE group and TACE alone group (OR=1.30, 95%CI: 0.78-2.18; *P*=0.31). There RCTs showed the incidence of liver dysfunction and the combined results suggested that the events of liver dysfunction were similar between these two groups (OR=1.00, 95%CI: 0.57-1.77; *P*=0.99).

### Results of publication bias

**Figure 9:**
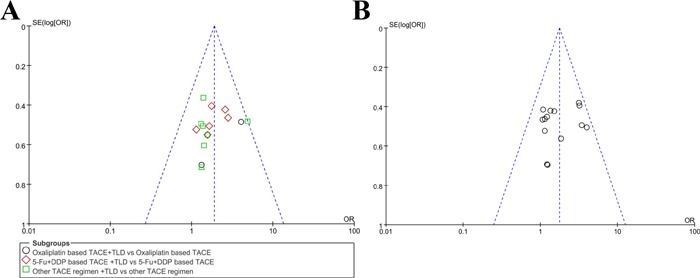
Funnel plot for publication bias (**A**, ORR; **B**, 1-year survival).

To assess the possibility of publication bias in ORR and 1-year survival rate, we introduced the Egg's test and funnel plot. The results showed that the symmetries were good in both funnel plots, suggesting that there was low risk of publication bias (Figure 9). The results of Egg's tests were 0.531 and 0.445 for ORR and 1-year survival rate. The risks of publication bias in DCR, parameters of cellular immunity, VEGF and adverse events were also evaluated, and the funnel plots were presented as supplementary figure. Overall, these funnel plots had good symmetry, suggesting that the results were less likely to be affected by publication bias.

**Table 4:** Evidence quality of combined effects

Endpoints	N	Combined effects	Quality of evidence
6-month survival	382	OR=1.79, 95% CI:1.02-3.15	moderate
12-month survival	1185	OR=1.76, 95% CI:1.38-2.24	moderate
18-month survival	212	OR=4.72, 95% CI:2.64-8.43	moderate
24-month survival	1111	OR=1.78, 95% CI:1.37-2.30	moderate
36-month survival	308	OR=2.32, 95% CI:1.27-4.21	moderate
ORR	1120	OR=1.89, 95% CI:1.48-2.42	moderate
DCR	986	OR=2.62, 95% CI:1.90-3.63	moderate
Reduction of VEGF	415	MD=-119.71, 95% CI:-135.75--103.68	moderate
Reduction of AFP	183	MD=-74.76, 95%CI:-90.25- -59.28	moderate
NK	164	MD=9.74, 95%CI: 5.60-13.89	moderate
CD_4_^+^/CD_8_^+^	64	MD=0.63, 95%CI: 0.45-0.80	moderate
Rash	211	OR=6.35, 95%CI: 2.75-14.68	moderate

Abbreviation: OR, odds ratio; MD, mean difference; ORR, overall response rate; DCR, disease control rate; VEGF, vascular endothelial growth factor; AFP, alpha-fetoprotein; NK, natural killer.

## DISCUSSION

Thalidomide has been widely used in clinical practice for treating several cancers including primary HCC. Several clinical trials suggested that thalidomide combined with TACE could significantly improve the clinical response rate, disease control rate, quality of life and survival rate in primary HCC, with tolerable adverse events. In this study, we systematically searched clinical studies on the topic of thalidomide combined with TACE versus TACE alone in patients with primary HCC, and used the synthesized data to determine the effect of thalidomide in improving the clinical efficacy of TACE. The combined results showed that thalidomide in combination with TACE had better ORR, DCR, quality of life and survival rates, and the decreases of AFP and VEGF were also significant, when compared with TACE alone. With regards to safety profile, administration of thalidomide significantly increased the incidence of drug rash, but not adverse events of gastrointestinal, myelo-suppression, and liver dysfunction.

The use of thalidomide has been proved to be beneficial in treating and delaying progression of several diseases, including HCC, multiple myeloma, and lung cancer. Several meta-analyses have evaluated the effectiveness of thalidomide in managements of multiple myeloma [17, 18, 55–61] and lung cancer [20]. These studies demonstrated that thalidomide maintenance therapy improved survival and disease control rates, which is in accordance with our main findings. The possible molecular mechanisms of thalidomide mainly includes anti-angiogenesis, inhibition of cytokines, such as TNF-á (tumor necrosis factor-á), bFGF (basic fibroblast growth factor) and VEGF(vascular endothelial growth factor) and modification of the expression of cell adhesion molecules [4, 17, 62]. The improvement in efficacy by adding thalidomide to TACE may be due to the anti-angiogenesis effect of thalidomide, as the VEGF level usually elevates after TACE [19, 37]. Compared with other anti-angiogenesis agents, especially sorafenib, thalidomide has been shown to be economical and practical benefits when combined with TACE and shows tolerable adverse events in primary HCC patients. Three meta-analyses [63–65] evaluated the effectiveness and safety of TACE plus sorafenib versus TACE alone for patients with unresectable HCC. The study of Liu et al. [63] reported that the DCR ranged from 18.4 to 91.2% in 10 noncomparative studies. Adding sorafenib to TACE may bring benefits for unresectable HCC in terms of TTP but not OS. In the study of Yang et al. [65], their meta-analysis of 6 studies including 1181 patients showed that the combination group of sorafenib and TACE had longer overall survival(HR=0.64, *p*<0.05) and TTP, and better response to treatment(RR=1.45, *p*<0.05) than TACE group. Fu et al. [64] also reported a similar conclusion that combination of sorafenib and TACE had survival and clinical benefits in patients with HCC, but associated with enhanced morbidity. In our included studies, the DCR ranged from 69 to 95%. The ORR data of these studies were available, and the combined data showed that application of thalidomide with TACE improved ORR in primary HCC patients. The DCR data from included studies were available, and the results showed a benefit of thalidomide for the improvement of DCR with non-significant heterogeneity. The combined benefits of using thalidomide in association with TACE in improving overall survival, progression free survival, and/or time to progression could not be calculated as relevant data was not provided by the included studies. However, by extracting data of survival rates at different follow-up, the synthesized results determined that thalidomide plus TACE had superior efficacy at 6, 12, 18, 24, 36 months, when compared with TACE alone. Indeed, several clinical trials[19, 66] and experimental studies [4, 24] also confirm the anti-tumor effects of thalidomide in HCC, and the results are encouraging.

As thalidomide shows effective outcomes in treating moderate and advanced HCC, whether tumor associated biomarker and angiogenesis related biomarker have roles in predicting its efficacy are being investigated [4, 25, 67]. In our meta-analysis, the changes of AFP and VEGF were reported by several included studies. Thalidomide with TACE reduced the serum level of AFP by a mean value of 141.95, better than TACE alone. Chen et al., [67] conducted a study to evaluate the clinical implications of AFP response in advanced HCC with thalidomide treatment. They included 42 patients for the final analysis, and defined AFP response as a 50% or greater reduction of AFP levels for 4 or more weeks during treatment. Radiographic response was determined by World Health Organization(WHO) criteria. The results showed that AFP response was obtained in 24% of patients, and it was independent prognostic factor for both PFS and OS. They concluded that AFP response after thalidomide therapy can more accurately reflect the biological response in HCC than radiographic response [67]. Another study conducted by Shao et al. [25], got a similar conclusion that early AFP response is a useful surrogate marker for predicting efficacy and prognosis in advanced HCC patients receiving thalidomide or sorafenib. With regard to VEGF, it has been proved to be a powerful pro-angiogenesis factor in several cancers including HCC [4, 68, 69]. In recent years, VEGF is also known as a prognostic biomarker in HCC patients treated with TACE or molecular targeted therapies [11, 70, 71]. In these studies, the level of VEGF before treatment is often used to predict clinical efficacy but not the changes of VEGF between prior- and post- treatments. Our results found that the mean level of VEGF was decreased by 123.64 in thalidomide plus TACE group than TACE group. These findings suggested that thalidomide could reduce the levels of AFP and VEGF, however, we could not determine whether the improved ORR, DCR and survival rates were associated with changed levels of AFP and VEGF. This needs well designed clinical trials and experimental studies to assess the relationship between changes of AFP and VEGF and prognosis and efficacy of thalidomide.

The quality of life in advanced HCC is always impaired and it gets worse when adverse events are emerged after treatments. How to enhance the anti-tumor effect of TACE without significantly affecting the quality of life and increasing the incidences of adverse events is extremely important. The pooled results showed that thalidomide treatment significantly improved quality of life in patients with HCC with tolerable safety events. Therefore, these points mentioned above may support the wide use of thalidomide in combination with TACE to obtain positive outcomes in terms of ORR, DCR, survival rates, quality of life and safety.

There were several limitations within this study. First, although all of the included studies were RCTs, differences in baseline characteristics, study design and clinical parameters of included patients were existed. For example, the ages, regions, performance status, and numbers of participants differed in included articles. Few studies reported detailed methods of randomization, and most of the studies did not report the method of blinding. The selective report bias may exist within the eligible studies, increasing the risk of publication bias. The different duration and doses of thalidomide treatment could increase the risk of heterogeneity, affecting the overall findings. Second, nearly all of the included studies were conducted in China, and they were small sample RCTs with limited number of participants. This could result in regional findings applicable for certain populations, decreasing the reliance of our results in clinical practice abroad. Even though, this meta-analysis was reliable and high quality as all of the selected studies were RCTs with relatively low risk of heterogeneity, and the results of this study could be used for guiding clinical treatment, especially for patients in China.

## CONCLUSION

In summary, thalidomide with TACE was associated with a significant improvement in the response rate and better survival rates in primary HCC patients. These results were especially noteworthy for patients without consideration of surgical resection or transplantation in China. The therapeutic effects of thalidomide observed in this meta-analysis may be due to the reduction in serum AFP and VEGF levels. Our study provided new evidence for treatment of moderate or advanced HCC. Well-designed, large sample, multi-center, RCTs were required to support our findings.

## MATERIALS AND METHODS

### Search strategy

Electronic databases including the Cochrane Library, Pubmed, Embase, CNKI, and Wan Fang were searched for eligible studies with the deadline of August, 2016. The systematically search was conducted using the following terms with different combinations: “thalidomide” and “TACE or transcatheter arterial chemoembolization” and “primary hepatic carcinoma, liver cancer or hepatocellular carcinoma”. The whole process of search was conducted by two reviewers, independently.

### Inclusion and exclusion criteria

The inclusion criteria for this study were as follows: (1) Study type: RCTs reporting clinical efficacy and safety of thalidomide combined with TACE versus TACE alone in treating patients with primary HCC, either in Chinese or English, regardless of blinding or allocation concealment; (2) Study subjects: patients were diagnosed as primary HCC with adequate evidence(histological diagnosis, imaging of liver enhanced MRI and/or CT )[8, 72], unsuitable for surgery, and Karnofsky score > 60, without limitation of sex, age, and race; (3)Interventions: thalidomide in combination with TACE versus TACE, thalidomide in combination with TACE and other treatments versus TACE and other treatments. The procedure and regimen for TACE were completely identical for both groups receiving TACE; (4)Endpoints: adequate and standard definition of primary endpoints included overall response rate(ORR), complete response(CR), partial response(PR), stable disease(SD) and progression disease(PD); adequate and standard definition of secondary endpoints were disease control rate(DCR), KPS score, survival rates at different times, changes of AFP and VEGF, and incidence of adverse events.

The exclusion criteria for this meta-analysis were as follows: (1) Study type: no clinical trials, no RCTs, retrospective studies; (2) duplicate publication; (3) un-reasonable design of methodology; (4) one-arm trial, case report, clinical experience, or review; (5) patients with poor performance status or unsuitable for TACE; (6) secondary or metastatic liver cancer; (7) no clear standards of diagnosis; (8) full-text without reporting required data.

### Study selection

The selection of RCTs was completed by two reviewers, independently. First, the title and abstract of identified studies were reviewed and those did not meet the inclusion criteria were excluded; Next, to identify remaining studies, the full-texts of possibly eligible studies were checked. If there was disagreement to anyone of possible included studies, a third reviewer was involved to decide include or not.

### Quality assessment

The methodological quality of included clinical trials were evaluated according to the standards of the Cochrane Reviewer Handbook 5.1.4. The risk of bias were classified as randomization, allocation concealment, blinding, incomplete data, selective data reporting and other potential sources of bias. The overall quality of included was defined as low, moderate, and high. Based on these standards, two reviewers independently assessed the bias of each eligible RCT. The inconsistent opinion on some RCT was solved by discussion or a third reviewer.

### Data extraction

The baseline data of each eligible study was extracted by two reviewers based on the previously prepared data extraction table. Data needed to be recorded was as follows: (1) general information such as title, first author, publication year, and source; (2) data about participants such as sex, age, diagnosis criteria, numbers of cases for each group, and lost to follow-up; (3) data about study design and performance such as trial type, duration of study and follow-up, interventions for different groups and measurements; (4) data of outcomes, including efficacy, survival rate, changes of KPS and VEGF, incidence of adverse events. To obtain essential data, contact with corresponding authors if necessity.

### Statistically analysis

All of the meta-analysis was performed using the RevMan 5.3 software. The methods used for statistically analysis were similar with previously published meta-analyses. Briefly, the heterogeneity between the included studies was analyzed by χ2 test. When the results of included studies were statistically homogeneous (*P*>0.1, I^2^<50%), the fixed effect model was used for meta-analysis. Otherwise, the random effect model was used for meta-analysis if the results of included studies were statistical heterogeneity (*P* <0.1, I^2^>50%). We also evaluated the possible factors that may contribute to heterogeneity, and subgroup analysis was performed based on these factors. In case of low-quality studies in meta-analysis, sensitivity analysis was used to test the stability and strength of the combined results. For dichotomous data, the pooled odd ratio(OR) were calculated with the 95% confidence interval (CI). For continuous data, the weighted mean differences(WMD) or Odds Ratio(OR) and their associated 95%CIs were calculated for every trial involved in the meta-analysis. We defined there was a statistically significant difference between combination group and TACE group if the *p*<0.05. The funnel plot was applied to detect possible publication bias, with the definition of *p*<0.1 as existing significant publication bias.

## SUPPLEMENTARY MATERIALS FIGURES


