# Supplementary material for: Thalidomide combined with transcatheter artierial chemoembolzation for primary hepatocellular carcinoma: a systematic review and meta-analysis
Source: Oncotarget. 2017 Mar 29;8(27):44976–93. doi: 10.18632/oncotarget.16689 (PMC5546534; doi:10.18632/oncotarget.16689)
Supplement: Supplementary file 1 [file oncotarget-08-44976-s001.pdf]

Thalidomide combined with thanscatheter arterial chemoembolization for primary hepatocellular carcinoma: a systematic review and meta-analysis

Supplementary Material

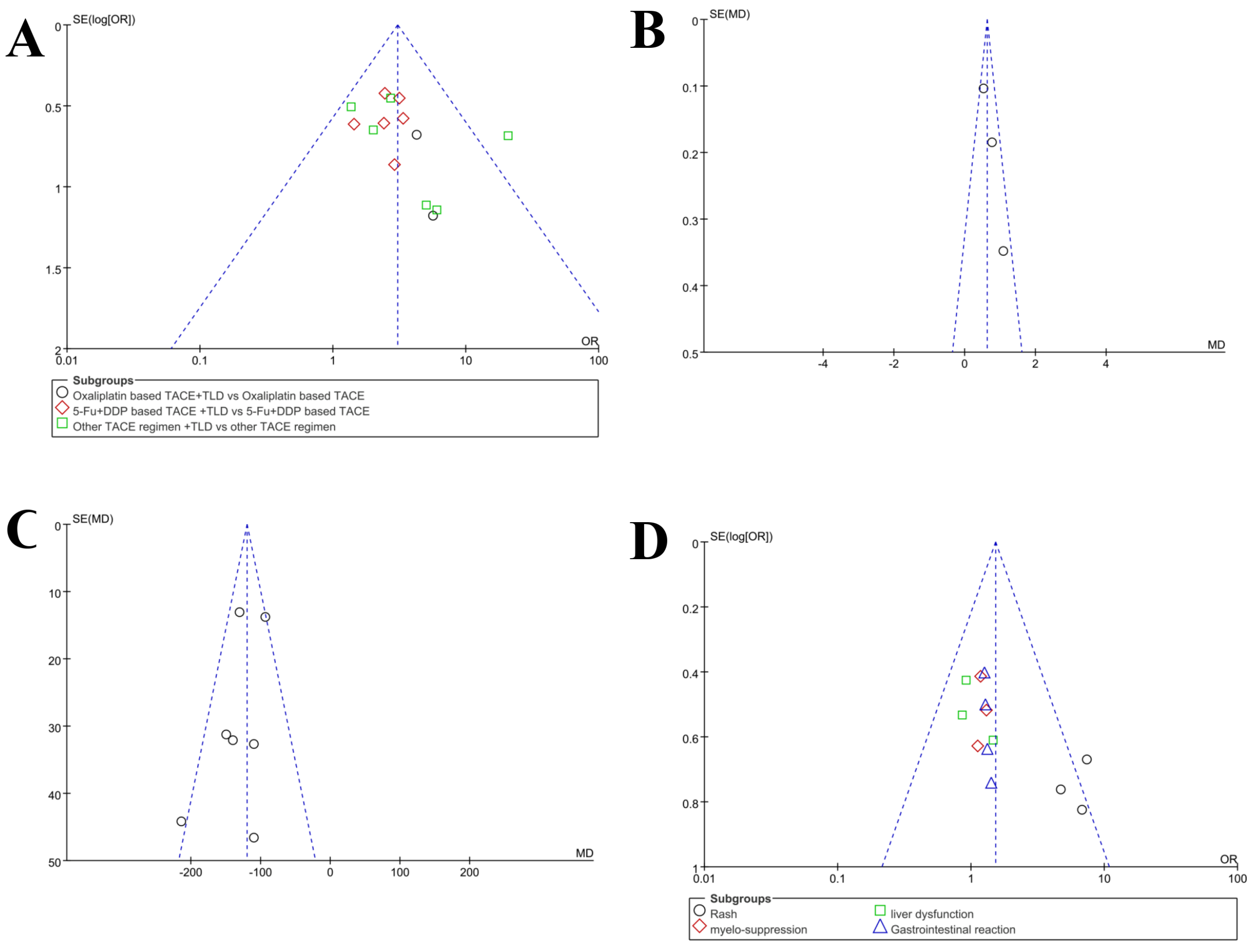

Supplementary Figure 1: Funnel plot for publication bias(A, DCR; B, CD4/CD8; C, VEGF; D, adverse events)
